# A meta-analysis of the efficacy of postoperative adjuvant radiotherapy versus no radiotherapy for extrahepatic cholangiocarcinoma and gallbladder carcinoma

**DOI:** 10.1186/s13014-020-1459-x

**Published:** 2020-01-15

**Authors:** Bixin Ren, Qi Guo, Yongqiang Yang, Lei Liu, Shaohua Wei, Wei Chen, Ye Tian

**Affiliations:** 1 Department of Radiotherapy & Oncology, The Second Affiliated Hospital of Soochow University, Suzhou, China; 2 Institute of Radiotherapy & Oncology, Soochow University, Suzhou, China; 3 Suzhou Key Laboratory for Radiation Oncology, San Xiang Road No. 1055, Suzhou, 215004 Jiangsu China; 4 Department of General Surgery, The Second Affiliated Hospital of Soochow University, Suzhou, 215004 China

**Keywords:** Extrahepatic cholangiocarcinoma, Gallbladder carcinoma, Adjuvant therapy, Radiotherapy, Meta-analysis

## Abstract

**Objective:**

The benefit of adjuvant radiotherapy (ART) for extrahepatic cholangiocarcinoma (EHCC) and gallbladder carcinoma (GBC) is unclear, with conflicting results from nonrandomized studies. We reported a meta-analysis to determine the impact of adjuvant radiotherapy on survival.

**Methods:**

PubMed, EMBASE, Cochrane Library and CNKI databases were searched to identify clinical trials of postoperative ART versus no radiotherapy for EHCC and GBC. The obtained data were analyzed using RevMan 5.3 and Stata 14.0 statistical software. Differences between two groups were estimated by calculating the odds ratio (OR) and 95% confidence interval (CI).

**Results:**

A total of 21 clinical trials involving 1465 EHCC and GBC patients were selected according to the inclusion and exclusion criteria and included in this meta-analysis. The meta-analysis showed the following: The 5-year overall survival (OS) rate was higher in the ART group than in the no radiotherapy group (OR = 0.63; 95% CI = 0.50–0.81, *p* = 0.0002). The 5-year OS rate was significantly higher for those with lymph node-positive disease (OR = 0.15; 95% CI 0.07–0.35; *p* < 0.00001) and margin-positive disease (OR = 0.40; 95% CI 0.19–0.85; *p* = 0.02) in the ART group than in the no radiotherapy group. ART had a tendency to bring benefit to the 5-year OS of patients with margin-negative disease but the difference was not statistically significant (OR = 0.57, 95% CI 0.30–1,07, *p* = 0.08). The local recurrence rate was significantly lower in the ART group than in the no radiotherapy group (OR = 0.54; 95% CI = 0.38–0.76, *p* = 0.0004), and there was no significant difference in the distant metastasis rate between the two groups (OR = 1.33; 95% CI = 0.95–1.87, *p* = 0.10).

**Conclusions:**

A meta-analysis of the existing study results showed that compared with no radiotherapy, ART is an effective postoperative treatment for EHCC and GBC.

## Introduction

Extrahepatic cholangiocarcinoma (EHCC) and gallbladder carcinoma (GBC) are rare, with approximately 12,360 new cases projected to occur in the United States in 2019 [1]. The prognosis of these cancers is typically poor, with a 5-year overall survival (OS) rate ranging from 5 to 19% [2]. The 5-year OS rate of patients who underwent surgery was reported to be 27 to 37% [3]. Obviously, surgical treatment can improve the 5-year OS. However, the risks of local invasion, lymph node metastasis and distant metastasis of EHCC and GBC are high, and these tumors are close to the complex anatomy of the porta hepatis, which limits surgery to some extent [2, 4–6]. Even after surgical treatment, the recurrence rate is very high; more than half of these patients experience recurrence after radical surgery, with local recurrence after resection being typically observed [7–10]. Although radiotherapy is an effective local treatment for eradicating potentially microscopic disease, the efficiency of adjuvant radiotherapy in EHCC and GBC patients is not clear.

Data supporting ART are sparse, and no consensus has been reached. Some studies have reported that patients can benefit from ART only early in the disease course but cannot benefit in the long-term [11–13]. At present, most published studies on ART for EHCC and GBC have been retrospective studies. Unfortunately, large randomized adjuvant trial evidence is difficult to obtain due to the relative rarity of EHCC and GBC. Therefore, we performed a systematic review and meta-analysis to investigate on the use of ART in EHCC and GBC patients and to clarify its effect on the 5-year OS and relapse of these patients, including the incidence of local recurrence and distant metastasis. To the best of our knowledge, no meta-analysis has yet evaluated the impact of ART on these outcomes in this patient population. Our study highlighted the benefit of long-term survival (5-year OS) and the effect of ART on local recurrence and distant metastasis. Confirmation of the effects of ART on this population and the identification of disease subgroups that would benefit from such a strategy will help guide the design of a prospective, randomized study for this rare disease. In addition, the results of this study can serve as a reference for clinicians.

## Methods

### Literature search strategy

Literature searches of PubMed, EMBASE, and Cochrane Library and CNKI (China National Knowledge Infrastructure) databases were performed from the date of inception to March 2019. Searches were limited to human studies. The main keywords used for the search were ‘extrahepatic cholangiocarcinoma’, ‘gallbladder cancer’ (or neoplasms), ‘bile duct cancer’ (or neoplasms), ‘radi.’ (radiotherapy, radiation), ‘chemoradi.’ (chemoradiotherapy, chemoradiation, radiochemotherapy), ‘adjuvant’ and ‘postoperative’.

### Selection criteria

Trials included tumors of the gallbladder and extrahepatic, perihilar, and distal bile ducts. In the experimental group, patients underwent surgery followed by ART, irrespective of concurrent chemotherapy. In the control group, patients underwent resection alone without ART. To avoid overlapping patient data in duplicate publications, registry analyses were excluded from this analysis.

### Statistical methods

Two authors extracted data independently to rule out subjective effects. The following details were extracted: study period, patient number and disease site (EHCC or GBC), nodal and resection margin status, chemotherapy use, radiation type and dosage, and ART toxicity. When reported, T stage and overall stage were collected. The use of concurrent chemotherapy (CT) was extracted when mentioned in the text. However, due to a lack of individual data and the fact that only a percentage of patients were treated with CT in some cohorts, it was not possible to statistically assess the impact of concurrent systemic treatments. The outcomes were 5-year OS, local recurrence rate and distant metastasis rate. When 5-year survival was not reported in the text, it was independently calculated from survival curves.

The relative frequency of OS at 5 years between the ART and no ART groups was expressed as the odds ratio (OR) and 95% CI. Data were extracted from the primary publications and entered into the meta-analysis using RevMan 5.3 and Stata 14.0 software. The level of heterogeneity between studies was evaluated with the Cochrane Q test and the I^2^ statistic. Egger’s regression test was used to assess publication bias.

## Results

### Studies

**Fig. 1:**
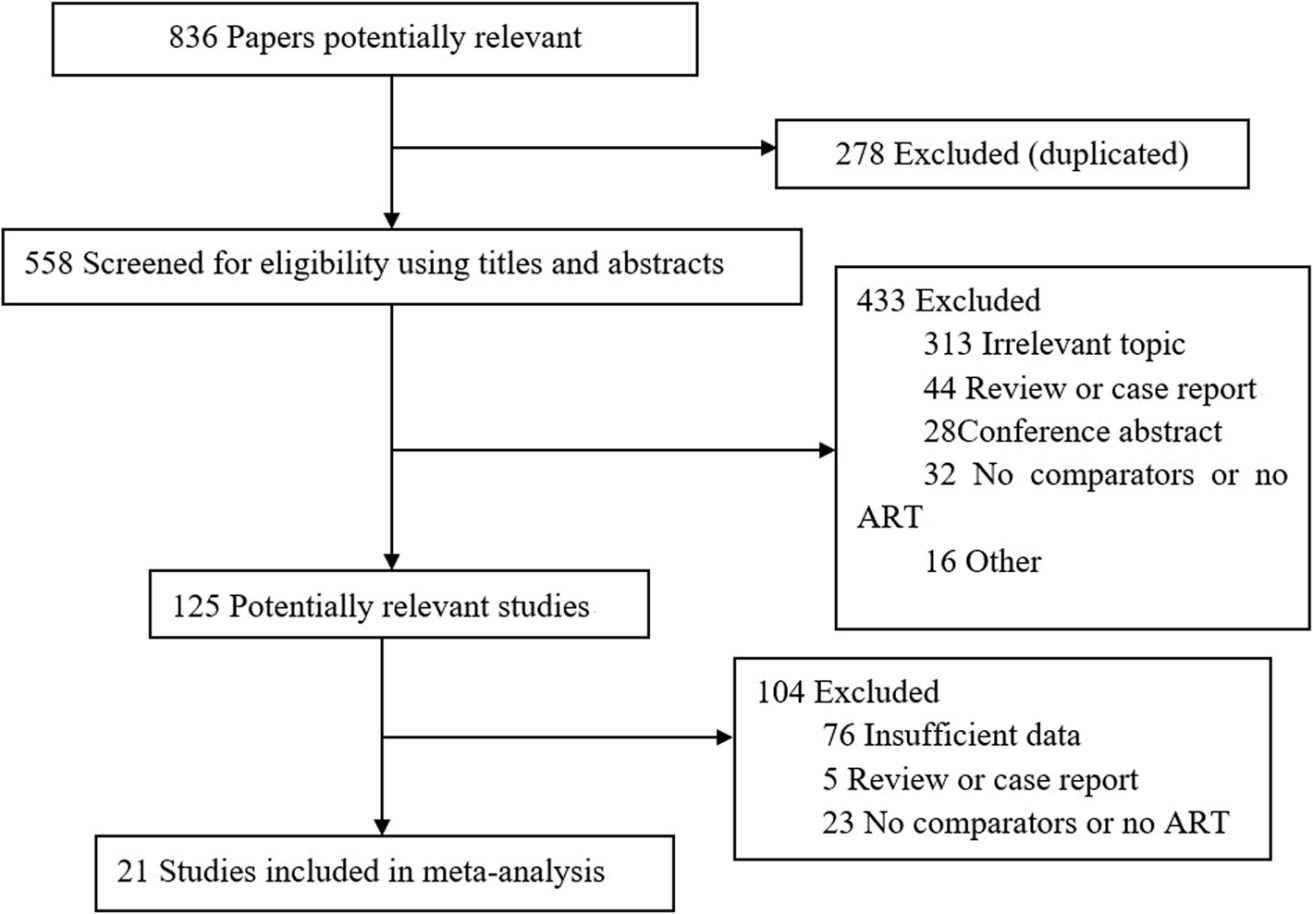
Flowchart of study inclusion

After screening for the inclusion criteria and reviewing the full texts of potentially eligible studies, 21 retrospective studies were identified. Figure 1 shows the flowchart of the literature search and selection process. These studies involved 1465 patients: 753 were treated with surgery alone, and 712 received ART. The detailed characteristics of the included studies are summarized in Tables 1 and 2. Some patients in the ART group also received concurrent chemotherapy, which was variable among the studies. The median external beam radiation therapy (EBRT) dose ranged from 37.5 to 52 Gy.

**Table 1:** Characteristics of the included studies

Author	Location	Study Period	Staging	Stage (S)	No. of Patients		Margin Positive (%)		Node Positive (%)	
					No ART	ART	No ART	ART	No ART	ART
Balalchandran et al. [14]	GBC	1989–2000	The 5th AJCC	I: 10.3%, II: 13.7%, III: 61.6%, IV: 12.8%	44	73	NR	NR	57	42
Borghero et al. [15]	EHCC	1984–2005	The 6th AJCC	I: 38.4%, II: 61.6%	23	42	0	64	13	28.3
Cheng et al. [16]	EHCC	1997–2002	The 6th AJCC	I: 30.7%, II: 12%, III: 49.3%, IV: 8%	34	23	NR	NR	NR	NR
Gerhards et al. [17]	EHCC	1983–1998	NR	I: 14%, II: 35%, III: 44%, IV: 5%	20	71	NR	NR	NR	NR
Gold et al. [18]	GBC	1985–2004	The 6th AJCC	I: 59%, II: 41%	48	25	0	0	30	70
Gu et al. [19]	GBC	2003–2013	The 7th AJCC	II: 48.5%, III: 41.5%, IV: 9.6%	39	39	0	0	NR	NR
Heron et al. [20]	GBC	1983–1997	NR	NR	13	13	NR	NR	NR	14
Hughes et al. [21]	EHCC	1994–2003	The 6th AJCC	II: 56%, III: 44%	27	35	7	26	37	82
Im et al. [22]	EHCC	2001–2010	The 6th AJCC	I: 31.3%, II: 58.9%, III: 9.8%	168	49	13.7	61.2	19.6	16.3
Itoh et al. [23]	EHCC	1994–2004	NR	I: 36.8%, II: 47.4%, III: 15.8%	10	11	25	82	NR	NR
Kim et al. [24]	EHCC	2000–2013	The 7th AJCC	I: 32.7%, II: 59.6%, III: 7.7%	33	19	30	74	27	42
Liang et al. [25]	GBC	1991–2006	The 7th AJCC	T1–2: 33.3%, T3–4: 66.7%	38	11	NR	NR	34.2	54.5
Lee et al. [26]	GBC	1994–2011	The 7th AJCC	T1: 24%, T2: 45%, T3: 23%, T4: 19%	83	62	11	12	11	19
Lindell et al. [27]	GBC	1991–1999	The 5th AJCC	I: 10%, II: 30%, III: 25%, IV: 35%	10	10	50	50	20	30
Meng et al. [28]	EHCC	1992–1997	NR	IVa	19	28	100	100	63.2	53.5
Pitt et al. [29]	EHCC	1988–1993	NR	NR	17	14	NR	87	NR	NR
Sagawa et al. [30]	EHCC	1980–1998	The 5th AJCC	I: 20.3%, II: 30.4%, III: 31.9%, IV: 17.4%	30	39	56.7	46.2	NR	NR
Todoroki et al. [31]	GBC	1976–1996	NR	IV: 100%	19	28	100	100	NR	NR
Todoroki et al. [32]	EHCC	1976–1999	AJCC	IVA: 100%	21	42	95.2	97.2	NR	NR
Wang et al. [33]	GBC	1985–2008	The 7th AJCC	T1–2: 58.9%, T3–4: 41.1%	44	68	9	37	18	63
Zlotecki et al. [34]	EHCC	1962–1993	NR	NR	13	10	NR	NR	NR	NR

**Table 2:** ART methods and toxicity

Author	ART	Dose	Adjuvant CT	Follow-up (m)	G3 acute toxicity	Late toxicity
Balalchandran et al. [14]	NR	NR	Yes	NR	NR	NR
Borghero et al. [15]	EBRT	45 Gy + (boost: 10 Gy)	Yes	31	3 gastrointestinal, 3 hematological	2 gastrointestinal ulcers, 1 biliary stenosis
Cheng et al. [16]	EBRT	50 Gy (44–56 Gy)	No	21	Nausea, vomiting	3 hepaticojejunostomy stenosis, 2 gastroduodenal bleeding
Gerhards et al. [17]	EBRT+/−IORT	EBRT: 46 Gy; EBRT+ IORT: 62 Gy	NR	28.8	abdominal pain, nausea, fever, diarrhea	Cholangitis, abdominal pain, ileus, high gastrointestinal bleeding, Roux-en-Y stenosis
Gold et al. [18]	EBRT	50.4 Gy	Yes	45	NR	NR
Gu et al. [19]	EBRT	50 Gy + boost: 5–10 Gy	Yes	20	No	No
Heron et al. [20]	EBRT+IORT	46 Gy	No	22	No	No
Hughes et al. [21]	EBRT	50.4 Gy (40–54 Gy)	Yes	41	NR	NR
Im et al. [22]	EBRT	50.4 Gy (41.4–54 Gy)	Yes	63	NR	NR
Itoh et al. [23]	EBRT	52.3 Gy (37.8–79.8 Gy)	No	32	NR	NR
Kim et al. [24]	EBRT	50.4 Gy (40–54 Gy)	Yes/No	24	No	2 duodenal ulcer
Liang et al. [25]	EBRT	50 Gy (40–64 Gy)	Yes/No	38.5	NR	NR
Lee et al. [26]	NR	NR	Yes	NR	NR	NR
Lindell et al. [27]	EBRT+/−IORT	EBRT: 40 Gy (IORT: 20 Gy)	Yes/No	NR	NR	NR
Meng et al. [28]	EBRT	52 Gy (45–62 Gy)	NR	30	No	No
Pitt et al. [29]	EBRT+IORT	NR	NR	NR	NR	Intestinal obstruction, hepatotoxicity, liver abscess
Sagawa et al. [30]	EBRT+IORT	EBRT: 37.5 Gy / EBRT+ IORT: 73.8 Gy	NR	32	NR	1 bile duct stricture, 1 venous hemorrhage
Todoroki et al. [31]	EBRT+/−IORT	EBRT: 40.0 Gy (IORT: 20 Gy)	NR	NR	NR	NR
Todoroki et al. [32]	EBRT+/−IORT	EBRT: 43.6 Gy (IORT: 21 Gy)	NR	30	1 liver necrosis	NR
Wang et al. [33]	EBRT	45 Gy + boost: 5.4 Gy	Yes	47.3	NR	NR
Zlotecki et al. [34]	EBRT	45 Gy (30–60 Gy)	NR	18	No	No

### Efficacy of ART in terms of 5-year OS

**Fig. 2:**
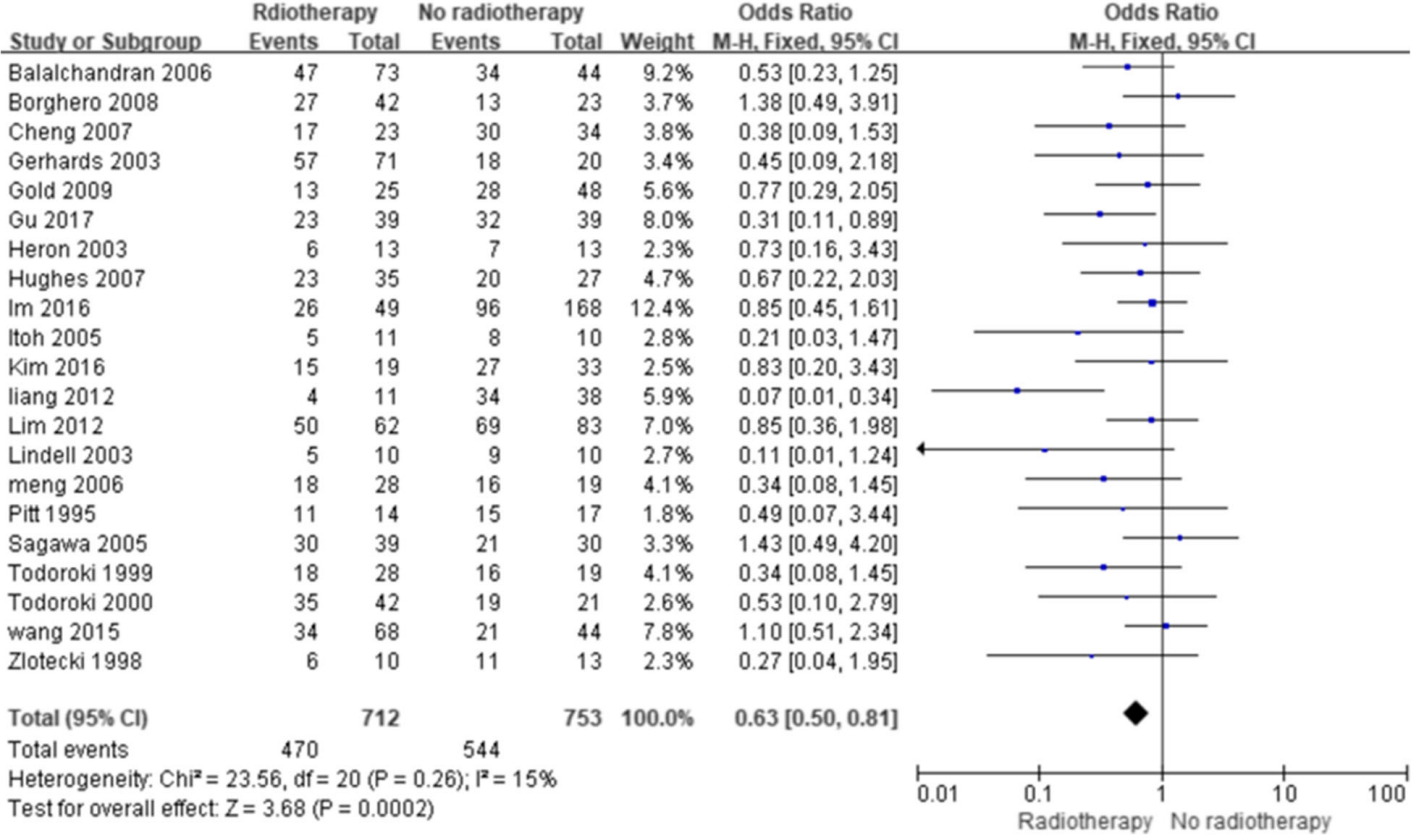
Forest plot of 5-year OS for the overall population

**Fig. 3:**
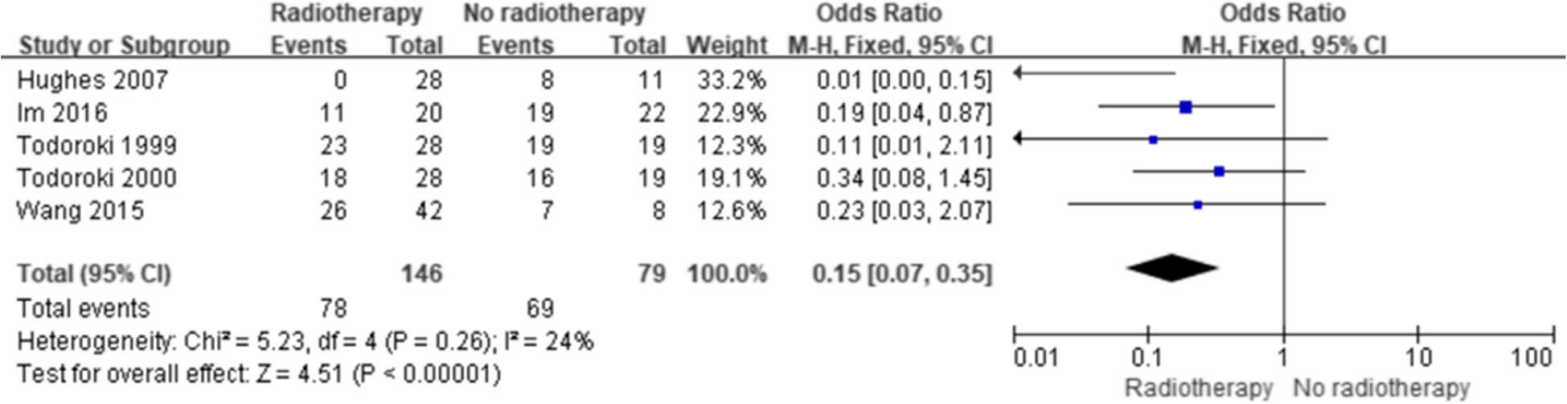
Forest plot of 5-year OS for lymph node-positive disease

**Fig. 4:**
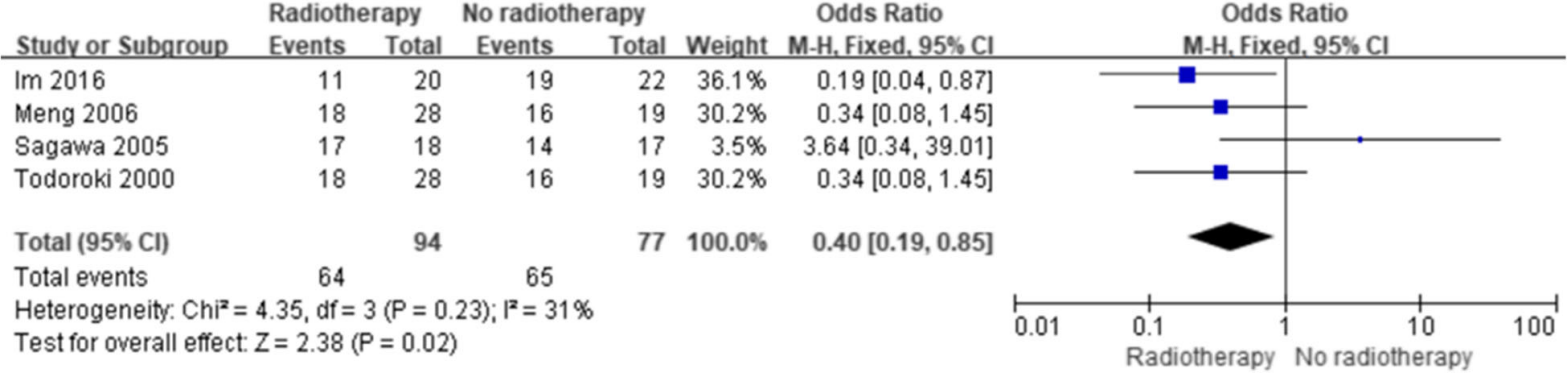
Forest plot of 5-year OS for margin-positive disease

**Fig. 5:**
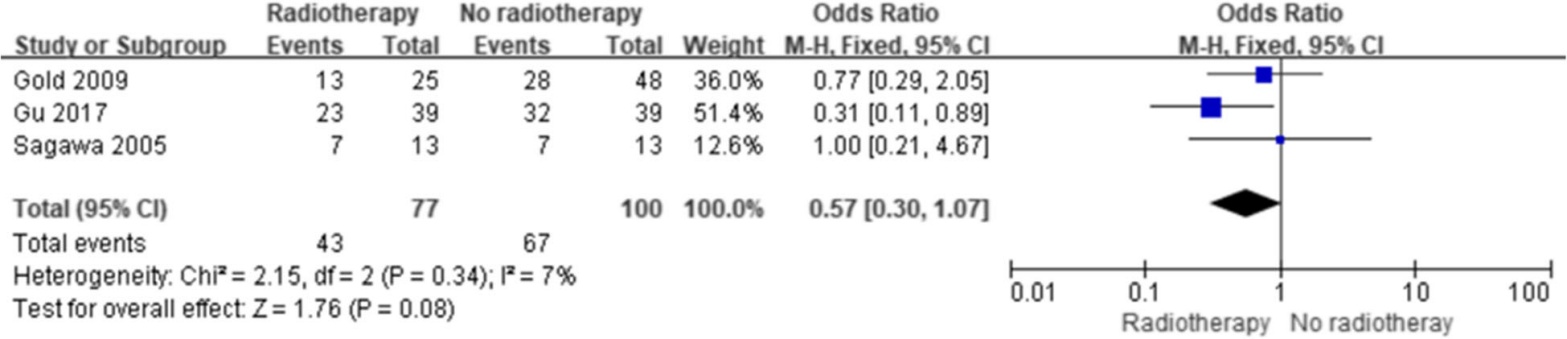
Forest plot of 5-year OS for margin-negative disease

The results showed a significant improvement in the 5-year OS with ART (with or without CT) compared with no ART (Fig. 2) (OR = 0.63; 95% confidence interval [CI] 0.50–0.81; *p* = 0.0002). No significant heterogeneity existed among the included studies (I^2^, 15%; *p* = 0.26). Five studies reported the 5-year OS of patients with lymph node-positive disease, and four studies reported the 5-year OS after margin-positive resection. Both analyses showed improvement in the 5-year OS with ART (OR = 0.15, 95% CI 0.07–0.35, *p* < 0.00001, Fig. 3; OR = 0.40; 95% CI 0.19–0.85; *p* = 0.02, Fig. 4, respectively). No significant heterogeneity existed among the included studies (I^2^, 24%, *p* = 0.26; I^2^, 31%, *p* = 0.23). Three studies reported the 5-year OS of patients with margin-negative disease, and the meta-analysis results showed that patients could gain a relative benefit from ART but the difference was not statistically significant (OR = 0.57, 95% CI 0.30–1.07, *p* = 0.08, Fig. 5).

### Efficacy of ART in terms of local tumor control

**Fig. 6:**
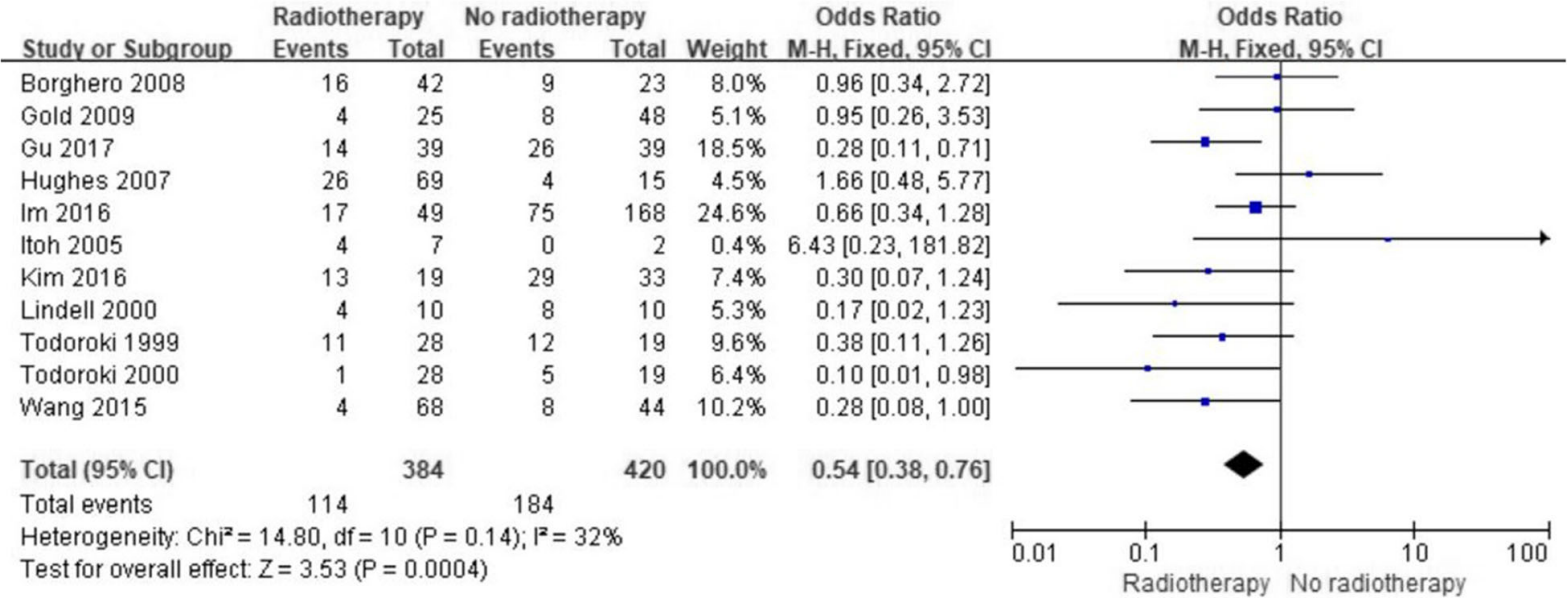
Forest plot of local recurrence

Eleven studies reported the influence of ART on local tumor control. Subsequent analysis of these studies revealed that ART significantly reduced the risk of local recurrence (OR = 0.54; 95% CI 0.38–0.76; *p* = 0.0004, Fig. 6). The included studies had no significant heterogeneity (I^2^, 32%; *p* = 0.14).

### Efficacy of ART in terms of distant metastasis

**Fig. 7:**
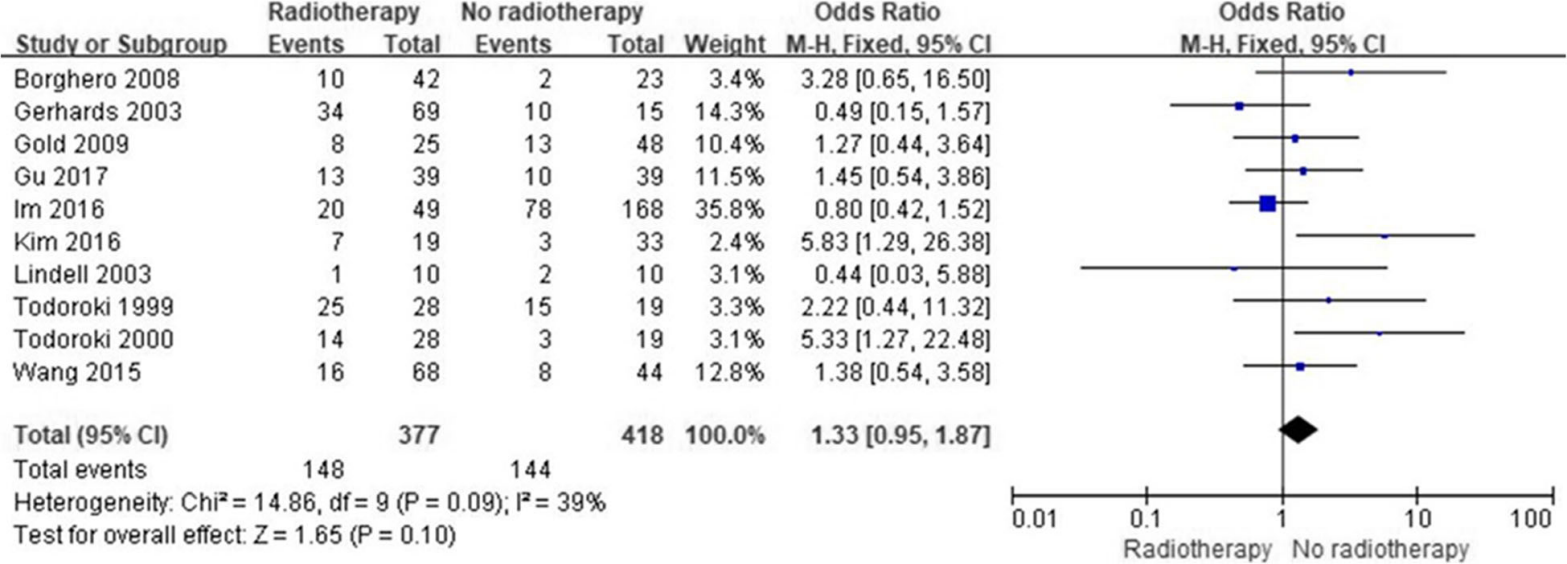
Forest plot of distant metastasis

Ten studies reported the impact of ART on distant metastasis. Pooled data showed a nonsignificant effect of ART compared with surgery alone on distant metastasis (OR = 1.33; 95% CI 0.97–1.87; *p* = 0.1, Fig. 7). Significant heterogeneity was found among the included studies (I^2^, 39%; *p* = 0.09). The studies by Kim et al. [24] and Todoroki et al. [32], who reported the least favorable effects of ART, were the main contributors to this heterogeneity. After exclusion of these two studies, the heterogeneity was not significant (I^2^, 0%; *p* = 0.5), but the effects of ART remained nonsignificant (OR 1.09; 95% CI 0.75–1.57; *p* = 0.66).

### Toxicities

The toxicities reported in the included studies are shown in Table 2. Acute and late toxicities were generally tolerable. The rates of grade 3 or higher acute toxicity (nausea, vomiting, diarrhea, bone marrow suppression, etc.) and chronic toxicity (gastrointestinal bleeding and ulcer, digestive tract obstruction, bile duct stricture, etc.) were low. A few patients required surgery because of severe toxic reactions, such as bleeding and stenosis.

### Literature publication bias evaluation

The publication bias of the included studies is shown in Table 3. The *p* value for publication bias regarding the overall 5-year OS was < 0.05, suggesting the presence of publication bias, and the remaining comparisons had a *p* value of > 0.05, indicating no significant publication bias. Egger’s regression test showed that publication bias existed in the 5-year OS. The scissor method showed that the combined results of the effect indicators before and after clipping were 0.468 (95% CI, 0.216–0.720) and 0.242 (0.011–0.474), respectively, using the fixed effects model and 0.523 (95% CI, 0.229–0.818) and 0.270 (− 0.058–0.599), respectively, using the random effects model. The results changed only slightly, indicating that the results in the literature were robust and that publication bias had little effect on the results.

**Table 3:** Evaluation of publication bias in the included literature

Evaluation index	Coef.	Std. Err.	t	P	95% CI
5-year OS	1.95	0.59	3.32	0.004	0.72–3.17
5-year OS of LN+	2.59	1.26	2.05	0.13	−1.43-6.60
5-year OS of R+	−5.97	1.60	−3.74	0.07	− 12.83-0.90
5-year OS of R-	−1.84	4.12	−0.45	0.73	−54.23-50.56
Local recurrence	−0.35	1.03	−0.34	0.74	−2.67-1.98
Distant metastasis	1.65	1.10	1.51	0.17	−0.87-4.16

## Discussion

This analysis included 21 studies (involving 1465 patients) that assessed the impact of ART on GBC and EHCC. In this comprehensive review and meta-analysis, we found that patients who received ART had a significantly better 5-year OS rate and lower local recurrence rate than those who did not receive ART. The results of our study were, to some degree, consistent with those of previous meta-analyses [35–37]. Horgan et al. concluded that adjuvant therapy was beneficial for high-risk biliary tract cancer patients, including those with lymph node-positive disease (HR, 0.49; 95% CI, 0.30–0.80) and those who underwent margin-positive resection (HR, 0.36; 95% CI, 0.19–0.68) [35]. Kim et al. reported that ART reduced the risk of death (HR, 0.54; 95% CI 0.44–0.67; *p* < 0.001) and recurrence (HR, 0.61; 95% CI, 0.38–0.98; *p* = 0.04) among patients with GBC [37]. However, being different from these studies, our study included several retrospective studies assessing the role of ART that were published recently, and we focused on the long-term (5-year) survival benefit of ART. In particular, we analyzed the effect of ART on local recurrence and distant metastasis. Our results suggest that ART is warranted and should be considered in prospective studies involving GBC or EHCC. Currently, there is no standard adjuvant treatment for patients with GBC or EHCC, and the controversy regarding whether the addition of ART improves OS in these patients has not yet been resolved [38–43]. Data are emerging from several prospective trials on the efficiency of adjuvant chemotherapy for GBC or biliary tract cancers, but prospective trials involving ART are scarce [44–46]. The BILCAP trial suggested that capecitabine, comparing with observation, can improve OS in patients with resected biliary tract cancer when used as adjuvant chemotherapy following surgery; however, this study did not meet its primary endpoint of improving OS in the intention-to treat population [44]. In a randomized phase III study conducted by Edeline J et al., adjuvant gemcitabine and oxaliplatin was found to offer no benefit in resected biliary tract cancer patients [45]. From the two trials, we can see that the benefit of adjuvant CT was unclear and patients may not receive much benefit from adjuvant CT, so it is necessary to explore the effect of ART with or without adjuvant CT. Recently, the SWOG 0809 trial of GBC and EHCC, a prospective single-arm trial, demonstrated that gemcitabine and capecitabine followed by concurrent capecitabine and radiotherapy had promising efficacy and the toxicity was acceptable [2]. Tran Cao et al. reported that ART was associated with a lower risk of death relative to surgery alone for patients with lymph node-positive GBC regardless of margin status (margin-negative resection: HR, 0.66; 95% CI, 0.52–0.84; margin-positive resection: HR, 0.54; 95% CI, 0.39–0.75) [47]. Lau et al. showed that the combination of surgery and radiation resulted in significantly longer survival than surgery alone (4.0 versus 3.7 years, *p* = 0.0004) [48]. However, some studies have shown that ART cannot benefit GBC and EHCC patients. For example, Leng et al. reported that ART did not improve the OS (22 vs 23 months, *p* = 0.978) of patients with curative intent resection of perihilar cholangiocarcinoma [49]. Among the studies in our meta-analysis, Borghero et al. [15] concluded that ART did not benefit patients, but the margins were negative in the observation group, and the margin-positive rate in the radiotherapy group was 64%. Therefore, the failure of radiotherapy to improve the 5-year OS may be due to the higher proportion of margin-positive patients in the radiotherapy group.

Some special points need to be noted in our study. Given the retrospective nature of these studies on GBC or EHCC patients who underwent surgery, it is reasonable to assume that a majority of the included patients who received ART were selected because of high-risk features, as our study showed that patients who received ART are more likely to have lymph node- and margin-positive disease. Thus, it is difficult to draw a conclusion on the best treatment for low-risk patients. In our analysis, only 3 studies reported the 5-year OS of patients with margin-negative disease, and we could not evaluate the effect of ART on lymph node-negative disease. Other details, such as the lymph node and margin status and the corresponding 5-year OS, were not always reported in the 21 studies. Thus, only five studies reported the effects of ART on patients with lymph node-positive disease, and four studies reported such data on those with margin-positive disease. Therefore, the results of the three analyses should be interpreted with extreme caution.

In terms of toxicity regarding adjuvant therapy, ASCO clinical practice guidelines for adjuvant therapy for resected biliary tract cancer indicate the risk of potential harm associated with radiation therapy for patients with GBC and EHCC [40]. However, in our included studies, the toxic reactions to radiotherapy were tolerable, and the incidence of acute toxicity above grade 3 (such as nausea, vomiting, diarrhea, bone marrow suppression, etc.) and chronic toxicity (such as peptic ulcer, gastrointestinal bleeding, digestive tract obstruction, bile duct stricture, etc.) was low. Severe toxic reactions, such as bleeding and stenosis requiring surgery, were rare.

This study also had several limitations. First and foremost, all the included studies were retrospective analyses and influenced by selection bias and treatment variations during the study period. However, all of these studies compared ART with no ART, and they all evaluated the same endpoints. Thus, we think that a meta-analysis is warranted to confirm the effect of ART on GBC and EHCC patients. Second, the number of patients included in each study was relatively small. Considering that registry study information is always incomplete and may overlap with institutional study information, we excluded registry studies. Third, due to the lack of relevant data, the efficacy of postoperative ART alone cannot be assessed, and because not all studies reported other details such as pathological condition and grade, it was difficult to draw conclusions regarding the best treatment for low-risk patients with early-stage, lymph node-negative disease.

## Conclusion

GBC and EHCC are rare and aggressive tumors for which there is no standard adjuvant treatment. Our study provides supporting evidence for the clinical application of ART in GBC and EHCC. The subgroup analysis in our study showed that patients at high risk due to lymph node-positive disease and margin-positive disease can benefit from ART. For patients with margin-negative disease, ART also had a trend to improve 5-year OS. We also conclude that ART can reduce the rate of local recurrence but does not affect the distant metastasis rate. Considering that no prospective trial has evaluated the effect of ART on GBC and EHCC, our study may contribute to the rational design of a prospective study and provide a reference for clinical treatment.

## Data Availability

All data generated or analyzed during this study are included in this manuscript.

## Abbreviations

ART: Adjuvant radiotherapy
CI: Confidence interval
CNKI: China National Knowledge Infrastructure
CT: Chemotherapy
EHCC: Extrahepatic cholangiocarcinoma
GBC: Gallbladder carcinoma
HR: Hazard ratio
OR: Odds ratio
OS: Overall survival
